# Assessment of knowledge and practice of nurses on initial management of acute poisoning in Dessie referral hospital Amhara region, Ethiopia, 2018

**DOI:** 10.1186/s12912-019-0387-2

**Published:** 2019-11-29

**Authors:** Ayele Mamo Abebe, Mesfin Wudu Kassaw, Nathan Estifanos Shewangashaw

**Affiliations:** 1 Primary author: Department of nursing, Debre Birhan Health Sciences College, 37 Debre Birhan, Amhara Ethiopia; 2 Department of nursing, College of Health Sciences, Woldia University, Woldia, Amhara Ethiopia

**Keywords:** Knowledge, Practice, Poisoning, Management, Nurses

## Abstract

**Introduction:**

Poisoning is a major health problem worldwide, and it causes significant morbidity and mortality. It is estimated that 350,000 people died worldwide from unintentional poisoning. The purpose of this study is to assess nurses’ knowledge and practice on the initial management of acute poisoning among nurses in Dessie referral hospital, Amhara region, Ethiopia.

**Methods:**

Hospital based cross sectional study was conducted from January 2018 to June 2018. Simple Random sampling technique was used to select the samples. Data was collected by using self-administered structured questioners. Data were cleansed, checked for completeness and entered into SPSS version 20 for analyses. Statistical measures of Central tendency, T-test and ANOVA were used in determining the association between independent and dependent variables.

**Results:**

Based on the given 13 items to assess the general knowledge of nurses on poisoning, the score ranges from 2 to 9 with the range of 7 with the mean score of 7.48(SD-0. 0.839) for the entire respondents that was 57.5% for the given items that was less than 75% which was unsatisfactory level of knowledge. The mean score of general knowledge on poisoning was high among nurses who had training on initial management of poisoning than those nurses who had not the training.

**Conclusion and recommendation:**

All Dessie referral nurses that participated in this study had unsatisfactory general knowledge on poisoning, knowledge on initial management of acute poisoning and self-reported practice. The major implication of these study findings on the health system is the importance of ensuring support to nurses’ health care services for early detection and management of poisoning.

## Introduction

Poison may be defined as any substance that can harm, kill by producing general or local destruction in the body [1–3]. It happens by the absorption of substances into the body to causing damage to the body cells [4, 5].

Poisoning is a major health problem worldwide [3, 6]. There are more than three million poisonings with 251,881 deaths occurring worldwide annually and 99% of the death occurs in developing countries [6]. In developed countries, acute poisoning is the leading cause for a visit to the emergency department among patients aged 2 to 30 years whereas, in developing countries, it is the second most common cause following infectious disease [7–9]. Death is higher in developing countries due to acute poisoning [10].

As WHO report, of all case deaths due to acute poisoning, Africa accounts 8% and Kenya 3.13% in 2015 [11]. According to a study in Ethiopia that revealed the highest proportion of poisonings happened in adults in which fatality rate was from 2.4–8.6% [12].

Acute drug poisonings account for nearly half of all poisonings reported [13–15]. Half a million people die annually due to poisoning [16]. WHO reported that acute poising is accounted 0.5% of intentional and unintentional injuries in Ethiopia, in 2012 [17–21]. Emergency department’s (ED) nurses should be equipped with the professional knowledge and skills to enable them to deal with a poisoned patient, and to assess the patients’ and family structure professionally [12, 22, 23].

According to the study done in Kenya, 51.5% of nurses were aware that women were more likely to take poison than men. About 64.7% of nurses had shown euthanasia when they got poisoning patient [24]. In Another study, the mean scores for acute poisoning knowledge practices ranged from 7.2 among certificate nurses to 7.4 for higher diploma & degree nurses [25].

Based on a study conducted in Egypt, all nurses in the studied sample (100%) had unsatisfactory knowledge level (< 75%) regarding detection and management of acute drug poisoning [26]. Similarly, in Hawassa study, All ED nurses that participated in the study had unsatisfactory knowledge (< 75%) [27]. since there was no study done in the study area, the findings of the study were very important to identify the factors of knowledge and practice of acute poisoning management.

## Method and material

### The study area and period

The cross-sectional study design was conducted in Dessie referral hospital, Amhara region, Ethiopia from January 2018 to June 2018. Dessie referral hospital is located 401 km to the North of Addis Ababa, the capital city of Ethiopia and 475 km far from Bahir Dar which is the capital city of Amhara region. The Source Population was all BSc and Diploma staff nurses who have been working in Dessie referral hospital.

### Sample size determination and sample procedure

To determine the sample size for the study, the following assumptions were considered:

*P* = 61.5% percentage of a proportion of nurses those have adequate knowledge poisoning management in Ethiopia from Hawassa study in 2016 was taken.

5% margin of error (d = 0.05).

## 10% for non-response rate

The Sample size was calculated by using the formula:

$$ \mathrm{ni}=\frac{{\mathrm{z}}^2\mathrm{p}\left[1-\mathrm{p}\right)}{{\mathrm{d}}^2} $$

Where n = sample size.

$$ {\mathrm{n}}_{\mathrm{i}}=(1.96)\times (1.96)\times 0.615(0.385)/(0.05)\ (0.05)\ \mathrm{for}\ \mathrm{population}.=363 $$

But our study population 256 total number of nurses in DRH is less than 10,000 the calculation was processed with reduction i.e. $nf= ni/\left(1+\frac{ni}{N}\right)=363/\left(1+\frac{363}{256}\right)$ = 150, and we add a non-respondent rate of 10% so final sample size was165.

### Sampling procedure

**Fig. 1:**
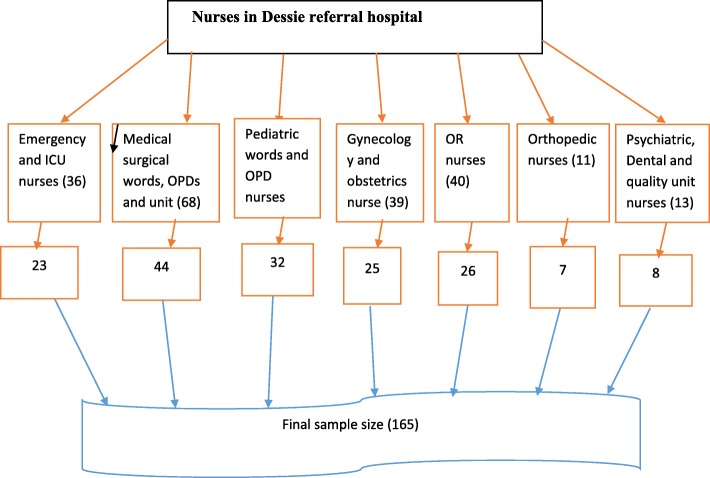
Sampling procedure participants in Dessie referral hospital

All 256 nurses working in the Dessie referral hospital was considered for the study. Participants were selected by using simple random sampling technique from each department based on proportion until the required sample size obtained (Fig. 1).

### Data collection procedure

The questionnaires were developed in English that includes all the relevant variables to meet the objective of the study. The data collected for this study was out by using a self-administered structured questionnaire.

### Data quality control

The questionnaire was pretested before the actual data collection by taking around 5% of the total calculated sample size in Kombolcha health center to check consistency, validity, and acceptability of the questioners. The Cronbach alpha result was 0.75 which showed the strong validity and reliability of the questionnaire.

### Data processing and analysis

The data was entered and analyzed through SPSS 20. A *P*-value less than 0.05 was cut point to say there was a positive association b/n the dependent and independent variables. Finally, the result was presented using frequency, percentage, tables, and graphs.

## Results

### Socio-demographic characteristics of the study participants

From a total of 165 participants recruited, 160 participated in the study while 5 unwilling to participate in the study, yielding the response rate of 97.0%. Among the 160 study participants, 96 (60.0%) were males. Majority of the respondents, 110(68.8%) were BSc holder (Table 1).

**Table 1:** The socio-demographic, experience and training of study participants Dessie referral hospital, North Eastern Amhara region, Ethiopia, 2018 (*n* = 160)

Variables	Frequency (*n* = 160)	Percent (%)
**Sex**		
Male	96	60.0
Female	64	40.0
Age		
20–29	122	76.3
30–39	28	17.5
40–49	7	4.4
> 50	3	1.9
Educational Level		
Diploma	49	30.6
Bsc	110	68.8
Msc	1	0.6
Length of experience		
< 3 years	112	70.0
4–10 years	46	28.8
11–15 years	1	0.6
> 15 years	1	0.6
Training on management of acute poising		
No	69	43.1
Yes	91	56.9

*Bsc* Bachelor of Science, *Msc* Master of Science

### Response of DRH nurses on their general knowledge of the poisoning

The item for which nurses displayed the highest knowledge level was on the definition of poison which was 138(86.3%).The item with lowest knowledge level was both classification of poison-based on its motive and cause, and alimentary signs and symptoms of acute poisoning at an early stage; which was homicidal poisoning 20(12.5%) and Dry mouth, abdominal pain and salivation 30(18.8%) respectively (Table 2).

**Table 2:** Response of DRH nurses on their general knowledge of poisoning, Dessie referral hospital, North Eastern Amhara region, Ethiopia, 2018

Knowledge on poisoning	Frequency correct response	Percent (%)
Poison is any substance capable of producing damage or dysfunction in the body by its chemical activity.	138	86.3%
Dose ingested and time of ingestion are not very necessary consideration when managing poisoning cases in ED.	48	30.0%
As an ED nurse it is always very important to treat the poison not the patient.	55	34.4%
The commonest cause of poisoning in developing countries is pesticide poisoning.	93	58.1%
Women are more likely to take deliberate poison in general population to commit suicide than men.	129	80.6%
Cause of poisoning among casualties attending any ED, according to motive and nature of use, can be classified as:		
I. Deliberate poisoning.	46	28.8%
II. Accidental poisoning.	65	40.6%
III. Homicidal poisoning.	20	12.5%
IV. Euthanasia poisoning.	56	35%
Alimentary signs and symptoms of acute poisoning during early stages include:		
I. Dry mouth, abdominal pain and salivation.	30	18.8%
II. Nausea, vomiting, hallucinations and convulsions.	63	39.3%
III. Coughing, cyanosis, hyperventilation and salivation.	61	38.1%
IV. Tachycardia, hypotension, diarrhea and breathlessness.	50	31.2%

### DRH nurses responses on initial management of acute poisoning practices

Hundred forty-eight(92.5%) of nurses answered correctly on the initial management of poisoning were on item said during organophosphate poisoning atropine should not be administered in any circumstance and the decision to perform gastro-intestinal decontamination 125(78.1%) and the volume of lavage fluid which was 102(63.7%). The least answered was an indication of gastric lavage which was 51(31.9%) (Table 3).

**Table 3:** DRH nurses responses on initial management of acute poisoning practices, North Eastern Amhara region, Ethiopia, 2018

DRH nurses responses on IMAP practice	Frequency Correct response	Percent (%)
1-In severe acute poisoning, maintaining adequate airway, respiration and circulation are always a priority.(T)	61	38.1
2-In case of organophosphate poisoning atropine should not be administered in any circumstance.(F)	148	92.5
3-Nearly all poisoning encountered in accident and emergency department have their specific antidote.(F)	105	65.6
4-The decision to perform Gastrointestinal (GI) decontamination should be based upon the specific poison(s) ingested, time from ingestion to presentation, and the predicted severity of the poison.(T)	125	78.1
5-Emesis is to be considered in an alert, conscious patient who has ingested a substantial amount of a toxic substance within 60 min of presentation.(T)	95	59.4
6-Activated charcoal can increase absorption of a wide range of poisons from the gastro-intestinal tract to the entire human system.(F)	71	44.4
7-Gastric lavage is indicated for patients who have ingested kerosene or corrosive substances within an hour of presentation.(F)	82	51.3
8-The effectiveness of gastric lavage increases as the time between ingestion and treatment increases.(F)	51	31.9
9-The volume of lavage fluid aspirated should approximate to the amount of fluid given.(T)	102	63.7
10-Patients presenting following ingestion of controlled/ slow released substances may benefit from decontamination even after a longer delay (e.g. more than 2–4 h).(T)	77	48.1

*IMAP* Initial Management of Acute Poising

### Self-reported nursing practice

**Fig. 2:**
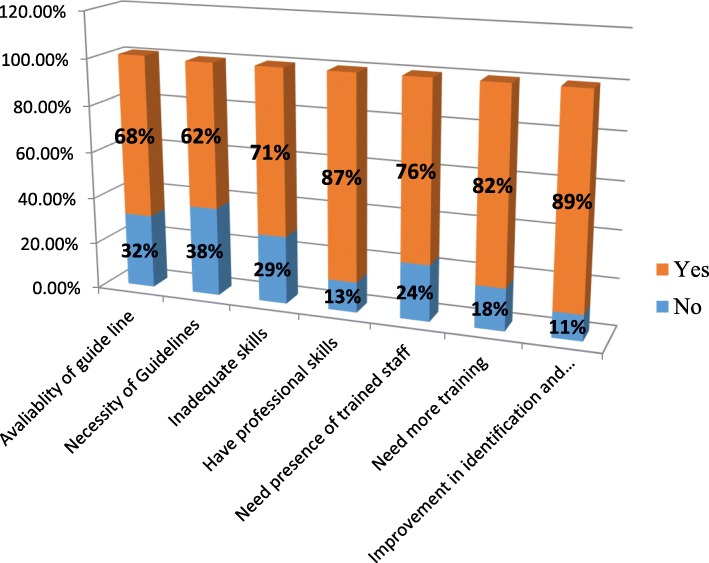
Figure that show self-reported nursing practice on initial management of acute poisoning among nurses in Dessie referral hospital in 2018 (*n* = 160)

Out of 160 nurses participated in the study, 49(32%) participants were responded that guidelines or flow charts were available at facility and 100(62%) nurses indicated that guidelines were necessary to assist in the management of poisoned casualty. One hundred twenty-one (76%) require trained or experienced staff while responding to poison-related cases (Fig. 2).

### General knowledge on poisoning, knowledge on initial management of acute poisoning and self-reported practice among nurses in Dessie referral hospital

Based on this study, the score ranges from 2 to 9 with the range of 7 with the mean score of 7.48(SD-0. 0.839) for the entire respondents which were 57.5%.This was less than 75% which was an unsatisfactory level of knowledge (Table 4).

**Table 4:** General Knowledge on poisoning, knowledge on initial management of acute poisoning and self-reported practice among nurses in Dessie referral hospital, North Eastern Amhara region, Ethiopia, 2018

			Range	
	Mean Score	Standard deviation	Minimum	Maximum
General Knowledge on poisoning	7.48	0.839	2	9
Knowledge on initial management of acute poisoning	7.28	0.891	5	10

### Educational status versus nurses’ general knowledge of poisoning

Diploma nurses score a mean score of 7.10(SD-0.549) which was the least score, BSc nurses score a mean of 7.65(SD-0.849) and MSc scoring a mean of 8.00 which was the highest score, the *p*-value was 0.001(Table 5).

**Table 5:** Educational status versus nurses general knowledge on poisoning Dessie referral hospital, north eastern Amhara region, Ethiopia, 2018

Educational status	Number	Mean (SD)	Range		ANOVA	
			Minimum	Maximum	p-value	F
Diploma	49	7.10 (0.549)	6	8	.001	7.939
Bsc	110	7.65 (0.849)	1	8		
Msc	1	8.00	8	8		

### Training versus nurses’ general knowledge of poisoning

The mean score of general knowledge on poisoning was high among nurses who had training on initial management of poisoning than those nurses who had not training (Table 6).

**Table 6:** Training versus general knowledge Dessie referral hospital, north eastern Amhara region, Ethiopia, 2018

Training	Number	Mean (SD)	Range		ANOVA	
			Minimum	Maximum	p-value	F
No	69	7.28 (1.056)	1	8	.006	7.608
Yes	91	7.64 (0.587)	7	8		

### Educational status versus knowledge on initial management of acute poisoning

Diploma nurses score a mean of 6.65(SD-0.8), BSc nurses score a mean of 7.54(0.75) and MSc nurse score a mean of 10 being the highest score, with the mean score of 7.28(SD-0.89) and *p*-value being 0.001 which shows there was strong statistically significant association between professional qualification and general knowledge of nurses on poisoning since p-value was < 0.05 (Table 7).

**Table 7:** Educational status versus knowledge on initial management of acute poisoning Dessie referral hospital, north eastern Amhara region, Ethiopia, 2018

Educational status	Number	Mean (SD)	Range		ANOVA	
			Minimum	Maximum	p-value	F
Diploma	49	6.65(.805)	5	9	.001	28.772
Bsc	110	7.54(.750)	6	10		
Msc	1	10.00	10	10		

## Disscussion

The purpose of the study was to asses both nurses knowledge on general and initial management acute poisoning, self-reported practices on the initial management of acute poisoning.

According to this study, the score ranges from 2 to 9 with the range of 7 with the mean score of 7.48(SD-0. 0.839) for the entire respondents that were 57.5% for the given items that was less than 75% which was unsatisfactory level of knowledge. Similar findings were reported in many international and local studies [25–28]. The unsatisfactory level of knowledge of nurses might be related to lack of training, absence of continuous supervision and evaluation.

This study had shown a strong positive association between nurses’ general knowledge of poisoning and educational status. The mean score of general knowledge on poisoning was increased across the group from Diploma holder nurse to MSc holder nurses. This finding was supported by studies done in Ethiopia, Kenya and Egypt [24–27]. This indicates that a higher level of professional nursing education boosted the general knowledge of nurses regarding the management of poisoning.

Unlike this study finding from Egypt, 2012, training on primary management of acute poisoning had a significant association with nurse’s general knowledge on the management of poisoning [26]. Nurses who took training on primary management of acute poisoning has a mean score of 7.64(SD-0.587) being the higher score and nurses who had no trained on initial management of acute poisoning had a mean score of 7.28(SD-1.056) being the lower score and *p*-value was 0.006 which was significant association with the dependent variable. This is reflecting that nurses can develop knowledge, skills, and understanding of patient care and treatment over time through sound educational base training.

Based on this study, the response of the nurses range between 5(min) to 10(max) the range was 5 and the entire mean score was 7.28(SD-0.891) which was 73% of the given items initial management of poisoning which shows nurses had unsatisfactory knowledge on initial management of acute poisoning. Still, this study finding was much higher than the finding from Egypt; Cairo which was shown nurses’ knowledge on the initial management of acute poisoning was 48.5% [26]. This discrepancy might be due to cross-country limitations of diagnostic tools and reporting biases, differences in socio-economic environments.

According to this study, there is no significant statistical association between length of experience of nurses and their knowledge on initial management of acute poisoning. This finding is in line with findings from both Egypt [26] and Kenya [25]. This might because of the majority (70%) of the study participants had a similar length of working experience.

Concerning about self-reported nursing practice, nearly to three fourth (68.9%) of participants underlined there was no standard guideline in their facility for the management of acute poisoning while 62% the study participants agreed on its necessity. Such gaps also reported from the study conducted in Hawassa which showed the availability of guideline was only 16% while its necessity was reported from 43% of the study participants [27].

Comparing this study results with facilities-based cross-sectional study done in Addis Ababa, we found our rates to be comparable across the board; inadequate skill (71% vs 65%), need presence of trained staff (76% vs 86%), and have professional skill (87% vs 86%,) which all revealed unsatisfactory nursing practice on the management of poisoning [27]. This difference may be due to nurses who worked in Addis Ababa had gotten more training on management of poisoned cases.

Eighty-two percent (82%) of this study participants reported they need more training or education pertaining to the management of poison, and 89% of participant nurses indicated that it was necessary to develop procedures that improve in the identification and management of acutely poisoned casualties seen at Dessie referral hospital. It is consistent with the study results from Ethiopia, Kenya and Egypt [24–27]. This low practice levels may be related to low knowledge level and loss of continuous education and training courses. Since this was cross-sectional study design establishing a temporal relationship between dependent and independent variable was impossible.

## Conclusions

All Dessie referral nurses that participated in this study had unsatisfactory general knowledge on poisoning, knowledge on initial management of acute poisoning and self-reported practice. Socio-demographic and work-related characteristics such as professional qualification and training had an impact on general knowledge and initial management of poisoning. The majority of Dessie referral hospital nurses required training related to emergency and poisoning.

## Data Availability

All data were available in this manuscript.

## Abbreviations

ABC: Air way Breathing and Circulation
BSC: Bachelor of Science
ECCN: Emergency and critical care nursing
ED: Emergency Department
GCS: Glasgow Coma Scale
KAP: Knowledge attitude and practice
MSC: Master of Science
WHO: world health organization
